# Hypoglycemic Efficacy of Docking Selected Natural Compounds against α-Glucosidase and α-Amylase

**DOI:** 10.3390/molecules23092260

**Published:** 2018-09-05

**Authors:** Jirawat Riyaphan, Chien-Hung Jhong, Shian-Ren Lin, Chia-Hsiang Chang, May-Jwan Tsai, Der-Nan Lee, Ping-Jyun Sung, Max K. Leong, Ching-Feng Weng

**Affiliations:** 1 Department of Life Science and Institute of Biotechnology, National Dong-Hwa University, Hualien 97401, Taiwan; mecurry@gmail.com (J.R.); 810613101@gms.ndhu.edu.tw (C-H.J.); d9813003@gms.ndhu.edu.tw (S-R.L.); 810413105@gms.ndhu.edu.tw (C-H.C.); 2 Neural Regeneration Laboratory, Neurological Institute, Taipei Veterans General Hospital, Taipei 11217, Taiwan; mjtsai2@vghtpe.gov.tw; 3 Department of Biotechnology and Animal Science, National Ilan University, Ilan 26047, Taiwan; dnlee@niu.edu.tw; 4 National Museum of Marine Biology and Aquarium, Pingtung 94450, Taiwan; pjsung@nmmba.gov.tw; 5 Graduate Institute of Marine Biotechnology, National Dong Hwa University, Pingtung 94450, Taiwan; 6 Department of Chemistry, National Dong Hwa University, Hualien 97401, Taiwan

**Keywords:** natural compound, α-glucosidase, α-amylase

## Abstract

The inhibition of α-glucosidase and α-amylase is a clinical strategy for the treatment of type II diabetes, and herbal medicines have been reported to credibly alleviate hyperglycemia. Our previous study has reported some constituents from plant or herbal sources targeted to α-glucosidase and α-amylase via molecular docking and enzymatic measurement, but the hypoglycemic potencies in cell system and mice have not been validated yet. This study was aimed to elucidate the hypoglycemic efficacy of docking selected compounds in cell assay and oral glucose and starch tolerance tests of mice. All test compounds showed the inhibition of α-glucosidase activity in Caco-2 cells. The decrease of blood sugar levels of test compounds in 30 min and 60 min of mice after OGTT and OSTT, respectively and the decreased glucose levels of test compounds were significantly varied in acarbose. Taken altogether, in vitro and in vivo experiments suggest that selected natural compounds (curcumin, antroquinonol, HCD, docosanol, tetracosanol, rutin, and actinodaphnine) via molecular docking were confirmed as potential candidates of α-glucosidase and α-amylase inhibitors for treating diabetes.

## 1. Introduction

Type II diabetes mellitus (TII DM) remains one of most prevalent health problems with incidence and mortality rates of about 4.95% and 4.00% worldwide [1]. Clinically, one type of anti-hyperglycaemia agents such as α-glucosidase inhibitors like Precose^®^ (acarbose) and Glyset^®^ (miglitol) are also employed to prolong the increases in post-meal blood glucose [2]. Nevertheless, these drugs are still liable to various adverse effects caused by chronic administration, including cardiovascular disease, lactate acidosis, hypoglycaemia, and gastrointestinal complaints [3,4,5]. The increasing prevalence of hyperglycaemia and negative clinical consequences of commercial anti-diabetic medicines have increased the demands to discover new drugs to control postprandial glucose levels [6] because high blood glucose leads the fluctuation of glycosylated hemoglobulin and further induces onset of diabetic complications. More importantly, seeking natural compounds as new anti-diabetic agents with little or no adverse effects becomes urgent issue.

It is believed that herbal medicine and alternative medication have smaller or fewer severe adverse effects [7]. Bismilachinone, smilachinin, chalcones derivatives, coumarins, ganoleucin derivatives, and curcumin elicit potential anti-diabetic activity [8,9,10,11,12]. However, the mechanism(s) of action of these anti-diabetic natural compounds are not fully understood, thus requiring an evidence-base evaluation [6]. Therefore, the anti-diabetic mechanism of action of some natural compounds of different origins, e.g., isovitexin, berberine derivatives, ellagic acid, achyrofuran derivatives, piplartine, and 3-deacetyl-3-cinnamoyl-azadirachtin, was studied through virtual screening via certain targets [13,14,15,16,17,18]. These studies have the potential to accelerate the discovery of the action mechanism of anti-diabetic natural compounds.

It is of critical importance to understand the putative molecular targets of those natural compounds. Interestingly, a previous report has evaluated the anti-diabetic efficacy of natural sources or folk herbal medicines by targeting α-amylase activity [19]. Recently, in silico approaches have been integrated in drug discovery to expedite the process and reduce the expenses [20]. In our previous study, top-ten compounds i.e., 16-hydroxycleroda-3,13-dien-16,15-olide (HCD), actinodaphnine, antroquinonol, berberine, catechin, curcumin, docosanol, quercetin, rutin, and tetracorsanol were selected from a pool of 47 natural compounds chosen as potential inhibitors against α-glucosidase and α-amylase activities through molecular docking and enzymatic activity assays [21]. As a confirmation the previous docking effort, this study further validated the potential of those ten selected compounds against α-glucosidase and α-amylase activities by in vitro glucose uptake assay and in vivo glucose and starch tolerance test with acarbose served as the positive control. This report is confirmed the hypoglycaemic efficacy of docking selected natural compounds against α-glucosidase and α-amylase in cell and mouse levels.

## 2. Results

### 2.1. The Cytotoxicity of Natural Compounds on the Caco-2 Cells

Caco-2 cells were employed to test the altered α-glucosidase activity of selected natural compounds in the next section. In this experiment in order to determine the optimal concentrations of test compounds for evaluating the lowering glucose benefit without causing any deterioration, the cytotoxic effects of selected natural compounds were firstly evaluated accordingly the cell viability measured in Caco-2 cells by the MTT assay.

**Figure 1:**
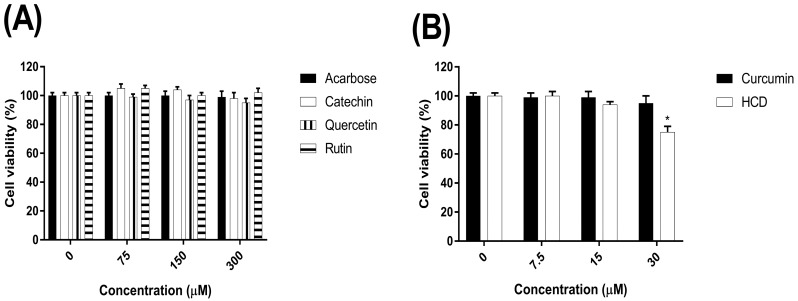

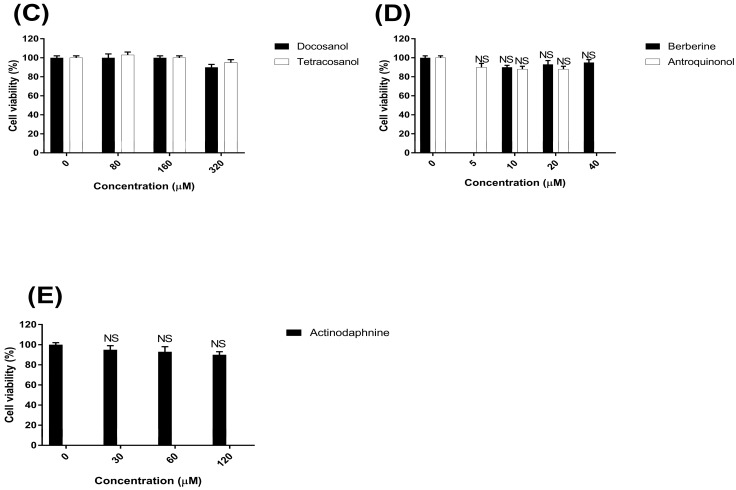
Cytotoxicity of selected compounds on Caco-2 cells. The cell viabilities were treated with various concentration of (**A**) acarbose, catechin, quercetin, rutin, (**B**) curcumin, 16-hydroxycleroda-3,13-dien-16,15-olide (HCD), (**C**) docosanol, tetracosanol, and (**D**) antroquinonol, berberine, and (**E**) actinodaphnine in Caco-2 cells measured via MTT assay and shown as the mean ± SD. * *p* < 0.05 when compared with the untreated control group (0 µM); NS, not significant.

The cells were incubated with various concentrations of test compounds for 24 h. When compared with the untreated control, the viabilities of cells were not significant difference (*p* > 0.05) (Figure 1B) in all tested compounds at various concentrations except the concentration of HCD at 30 µM (*p* < 0.05) (Figure 1A,C,D,E), suggesting that the certain concentrations of selected natural compounds were not cytotoxic and plausible to further investigate their inhibitory effects of α-glucosidase activity.

### 2.2. Inhibitory α-Glucosidase Activity of Selected Natural Compounds in Cells

**Figure 2:**
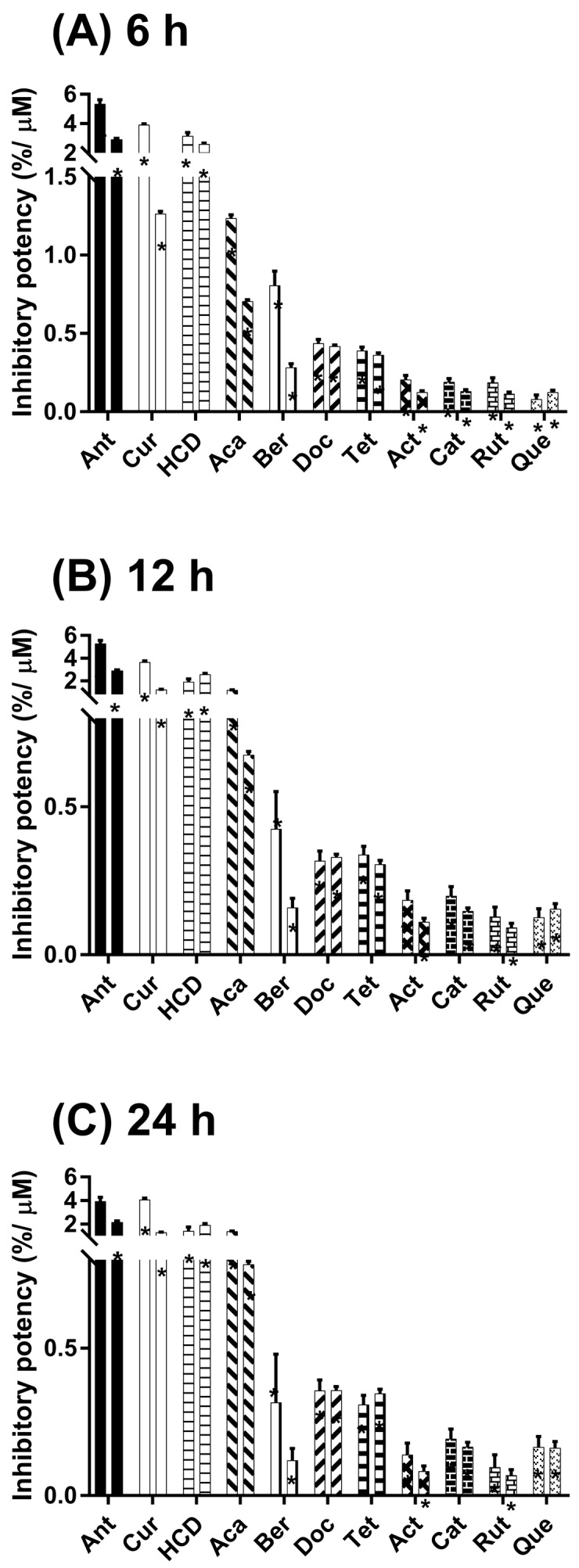
Inhibitory effect of test compounds on the in vitro maltose digestion. Caco-2 cells were treated with test compounds (acarbose (Aca) 40 or 80 μM; antroquinonol (Ant) 5 or 10 μM; catechin (Cat) 40 or 80 μM; quercetin (Que) 40 or 80 μM; actinodaphnine (Act) 40 or 80 μM; curcumin (Cur) 10 or 40 μM; docosanol (Doc) 40 or 80 μM; tetracosanol (Tet) 40 or 80 μM; rutin (Rut) 40 or 80 μM; berberine (Ber)10 or 40 μM; 16-hydroxycleroda-3,13-dien-16,15-olide (HCD) 5 or 10 μM) and maltose for (**A**) 6 h, (**B**) 12 h, and (**C**) 24 h prior to analyze glucose concentration in culture medium. The data are presented as mean ± SD. * *p* < 0.05 as compared with maltose alone.

To measure their inhibitory efficacy of α-glucosidase activity, various concentrations of test compounds were incubated with maltose for various times in Caco-2 cells, followed by determining the glucose concentration in the culture medium. The inhibitory potency of test compounds in Caco-2 cells at 6-h incubation (Figure 2A) was associated with the measurement of α-glucosidase activity in test tube enzymatic assay of our previous study [21]. Additionally, the tendency of this inhibition was dependent on the concentrations of test compounds. Subsequently, the α-glucosidase inhibition of test compounds in Caco-2 cells was extensively performed to a 12-h incubation (Figure 2B). Furthermore, some of test compounds such as catechin, quercetin, curcumin, docosanol, and tetracosanol sustainably inhibited the α-glucosidase activity after a 24-h incubation (Figure 2C). These results suggest that test compounds unequivocally exhibit inhibitory effects of α-glucosidase in Caco-2 cells.

### 2.3. Hypoglycemic Effects in Oral Administration of Natural Compounds in Mice

**Figure 3:**
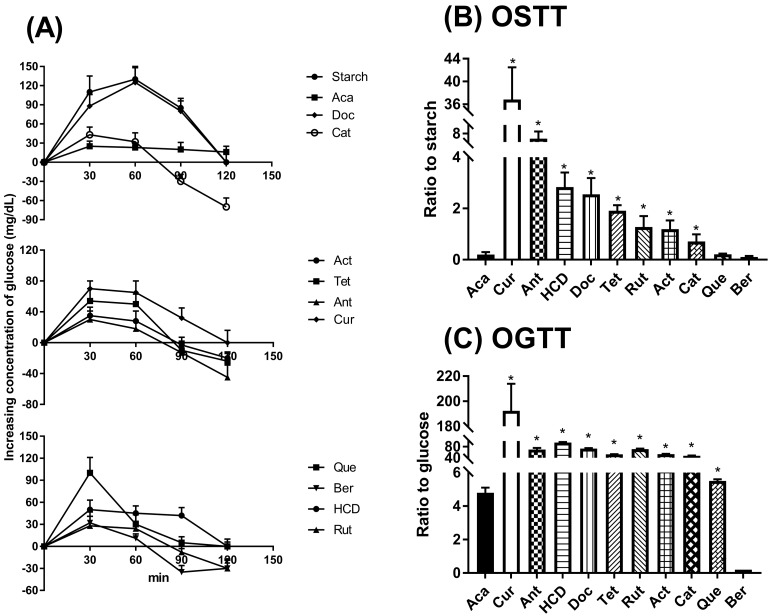
Hypoglycemic effects on oral administration of glucose or starch with acarbose or natural compounds in mice. 4.0 g/(kg·B.wt.) of glucose (OGTT) or 2.5 g/(kg·B.wt) of starch (OSTT) mixed with single dose of natural compounds (acarbose (Aca) 17 mg/kg, curcumin (Cur) 0.3 mg/kg, 16-hydroxycleroda-3,13-dien-16,15-olide (HCD) 3 mg/kg, docosanol (Doc) 4 mg/kg, tetracosanol (Tet) 4 mg/kg, antroquinonol (Ant) 1 mg/kg, berberine (Ber) 100 mg/kg, catechin (Cat) 6 mg/kg, quercetin (Que) 60 mg/kg, actinodaphnine (Act) 5 mg/kg, and rutin (Rut) 4 mg/kg) were oral gavaging followed by blood sampling every 30-min interval until 120 min, respectively. (**A**) Increasing blood sugar as compared with initial; (**B**) The ratio to starch; and (**C**) The ratio to glucose was calculated and compared. * *p* < 0.05 when compared with acarbose during glucose or starch given.

To test the hypoglycemic effects of selected compounds, an in vivo oral glucose tolerance test (OGTT) and an oral starch tolerance test (OSTT) were carried out. Among the ten test compounds in OGTT, the results illustrated that curcumin, HCD, antroquinonol, and berberine exhibited similar curves when compared with acarbose (*p* < 0.05, Figure 3A,C). Of all the natural compounds in OSTT, only curcumin, HCD, berberine, and quercetin exhibited similar curves when compared with acarbose (*p* < 0.05), suggesting that none of these natural compounds is no stronger than acarbose in hypoglycemic effects (Figure 3B). After comparison with the glucose lowering concentration of the reference drug acarbose, the selected natural compounds were categorized into four groups after the conversion of potency into the fold-increases with respect to acarbose set as one: namely Groups 1–4, whose increases are >37.7-fold; between 10.9–37.7; between 4.4–7.2; and between 0.7–1.2, respectively. The classification results are displayed in Table 1. These results further confirmed that the previously selected natural compounds via docking possess the inhibition of α-glucosidase and α-amylase against hyperglycemia at the cellular and animal levels.

## 3. Discussion

There was no significant difference between berberine and acarbose in the potency for lowering glucose, implying the similar hypoglycaemic abilities of both compounds, which, in turn, were less potent than the other test natural compounds (Table 1). Berberine is the main active constitute of rhizome of *Coptis chinesis*, which has been traditionally used for a long period of time in China to treat diabetes [22]. Meanwhile, berberine could reduce blood-glucose levels and regulate lipid metabolism in normoglycemic human subjects [23,24]. The lower potency of berberine than quercetin in OGTT and OSTT unequivocally indicts its weaker antihyperglycemic activity.

Acarbose, which is a well-known inhibitor of α-amylase, α-glucosidase, and cyclomaltodextrin glucanotransferase, is dependably used as the treatment of TII DM [25]. Our in vitro cell test and in vivo OGTT and OSTT results indicated an inhibitory effect of carbohydrate digestion in all selected natural compounds except berberine and quercetin. Of the test compounds, curcumin and antroquinonol were found to have the highest hypoglycaemia potency against α-glucosidase and α-amylase when compared with the reference drug acarbose (Table 1). Curcumin is the major constituent of turmeric (*C. longa*), that various reported activities including anti-tumour, anti-malaria, immunomodulation, and anti-diabetes effects [26]. In fact, the anti-diabetic effects of curcumin are exerted by activating the peroxisome proliferator-activated receptors γ (PPAR γ) signalling pathway and upregulating GLUT4 translocation [12], increasing insulin secretion and sensitivity, regulating glucagon synthase kinase 3 (GSK-3) [27], and inhibiting DPP-4 activity [28]. Previously, curcumin and its derivatives (curcuminoids) had been proved as α-glucosidase inhibitors in an enzymatic manner [29]. The present study was the first evidence not only confirming our previous in silico and in enzymatic results [21] but also extending the treating potential of curcumin into in the vitro cellular assays and in vivo mouse level.

Antroquinonol, which is an active triterpenoid isolated from mycelium of *A. cinnamomea*, has shown anti-diabetic efficacy by inhibiting DPP-4 activity, subsequently leading to hypoglycaemic activity [30]. It was observed in this study that antroquinonol exerted inhibitory activity on carbohydrate digestion in vitro and in vivo, indicating a new approach for its hypoglycaemic mechanism. Several current studies have reported that rutin, quercetin, catechin, and a variety of flavonoids isolated from *T. sinensis*, could reduce blood sugar levels in animals, plausibly due to α-amylase inhibition [31]. Obviously, it has been previously demonstrated that the alkaloids can exert higher inhibitory activity than flavonoids against α-glucosidase, aldose reductase, α-amylase, and lipase [32]. Rutin has been proved to promote insulin receptor kinase activity resulting in enhanced glucose transporter 4 translocation and glucose uptake [30]. Rutin, which did not show α-amylase and α-glucosidase inhibitory activities in the direct enzymatic assays of previous studies [21,33], decreased the blood sugar concentrations at 30 min and increased the blood glucose concentration to 111.6 mg/dL at 30–60 min after oral starch administration and showed inhibition in in vitro cell tests. It seems possible to postulate that the animal test is more reliable to confirm the results of in silico experiments, enzyme assays, and in vitro cell tests [34]. Additionally, our study has indicated that rutin could improve glucose uptake via enhancing insulin receptor kinase activity [35]. Taken together, this in vivo hypoglycaemic potency of rutin might be due to some other signalling pathways unrelated to amylase and glucosidase activity.

In in vitro and in vivo tests, quercetin showed lower inhibitory potency among the ten candidates and acarbose, which is consistent with the results of a direct α-amylase activity assay. Interestingly, the inhibitory potency of quercetin increased with incubation time in cell tests, which might suggest a delayed amylase-inhibiting reaction. In previous studies, the enzymatic inhibitory activity of quercetin was inconclusive. When compared with rutin, high or low inhibitory activities were occasionally reported [32,33]. Furthermore, the combination of quercetin and curcumin as antioxidants is more effective to control blood glucose levels in TII DM [36]. Nevertheless, it is necessary to carry out more detailed in vivo studies to characterize the hyperglycaemia models that demonstrate the anti-diabetic efficacy displayed by various natural compounds.

## 4. Materials and Methods

### 4.1. Chemicals

The chemicals used in this study were all analytical grades and purchased from Sigma Aldrich (St. Louis, MO, USA). The reagents and media for cell culture were obtained from Thermo-Fisher (Waltham, MA, USA). Preparations of the ten tested natural compounds were described in our previous study [21]. Natural compounds were dissolved in Dulbecco’s minimum essential medium.

### 4.2. Cell Culture and Cell Viability Assay

Caco-2 cells were cultured in T-75 tissue culture flask contained DMEM with 20% fetal bovine serum (FBS) and 1% penicillin-streptomycin (PS) at 37 °C, 5% CO_2_ in CO_2_ incubator (Thermo-Fisher, Waltham, MA, USA). 1 × 10^4^ cells/well of Caco-2 cells were performed to measure the cell viability followed previous protocol with modification [37].

### 4.3. In Vitro α-Glucosidase Activity Assay in Cells

*In vitro* α-glucosidase inhibitory assay was described in the previous study with slight modification [38]. Briefly, Caco-2 cells (1 × 10^4^ cells/well, 96-well) reached 100% confluence and washed 3 times with phosphate buffer saline (PBS) and added with various concentrations of test compounds (acarbose; catechin; quercetin; actinodaphnine; docosanol; tetracosanol and rutin 40 or 80 μM, respectively; antroquinonol 5 or 10 μM; curcumin 10 or 40 μM; berberine 10 or 40 μM; HCD 5 or 10 μM) mixed with 28 mM maltose in glucose-free medium for sampling at various times including 6, 12, and 24 h to measure the glucose concentration. The glucose concentration of the culture medium was determined using the glucose oxidase method. Briefly, the glucose standard curve was simultaneously generated as 0.0–1.0 nmol/well with a glucose assay buffer. The whole reaction was incubated at 37 °C for 30 min. The claret color measured at 490 nm is proportional to the original glucose concentration [39]. The inhibitory potency of each treatment was calculated from glucose generating rate (GGR) by following equations:

Inhibition percentage = [GGR (control) − GGR (treated)/GGR (control)] × 100%(1)

Inhibitory potency = Inhibitory percentage/natural compounds concentration (μM)(2)

### 4.4. Animals

8-week-old male C57BL/6 mice were obtained from the National Laboratory Animal Center (Taipei, Taiwan) and kept at controlled environmental conditions at room temperature (22 ± 2 °C) and humidity (55 ± 10%). The 12 h light/dark (0600–1800) cycle was maintained throughout the study. The animals had free access to a commercial diet and were provided water ad libitum. Animal experimental protocols were followed as per the “Guide for the Care and Use of Laboratory Animals” of National Dong-Hwa University approved by the National Dong-Hwa University Animal Ethics Committee.

### 4.5. Oral Glucose and Starch Tolerance Test (OGTT and OSTT)

The protocols of OGTT and OSTT followed the previously described with slight modification [40]. The used dosages of glucose and starch were 4.0 g/(kg·B.wt) and 2.5 g/(kg·B.wt), respectively. These tested glucose and starch amounts were determined by pretest and found more obvious to observe the difference among treatment during OGTT and OSTT. The dosage of different natural compounds is based on (1) literature review (2) cytotoxicity test and (3) pilot study. The dosage of natural compounds also enlisted: acarbose 17 mg/(kg·B.wt), curcumin 0.3 mg/(kg·B.wt), HCD 3 mg/(kg·B.wt), docosanol 4 mg/(kg·B.wt), tetracosanol 4 mg/(kg·B.wt), antroquinonol 1 mg/(kg·B.wt), berberine 100 mg/(kg·B.wt), catechin 6 mg/(kg·B.wt), quercetin 60 mg/(kg·B.wt), actinodaphnine 5 mg/(kg·B.wt), and rutin 4 mg/(kg·B.wt) The inhibitory potency was calculated from the inhibition ratio contributed by each dose of selected natural compounds:

Inhibitory potency = {1 − [AUC (treated)/AUC (control)]} natural compounds dose (mg/(kg·B.wt))(3)

### 4.6. Statistical Analysis

All data were expressed as means with standard deviations (mean ± SD) and the data were analyzed using one-way ANOVA with Tukey’s test. Statistical significance was defined as *p* < 0.05. All statistical procedures were performed with GraphPad Prism version 7.04 (GraphPad Software, Inc., La Jolla, CA, USA).

## 5. Conclusions

Taken all data together, among the ten selected natural compounds, seven (curcumin, antroquinonol, HCD, docosanol, tetracosanol, rutin, and actinodaphnine) are better therapeutic agents for lowering blood glucose than acarbose, which is a marketed drug. Furthermore, this study has paved the way for further research searching for natural sources to suppress the on-target enzymes, namely α-glucosidase and α-amylase, for the alternative option of TII DM therapy with little or negligible adverse side-effects.

## Figures and Tables

**Table 1:** The efficacy (fold change) of natural compounds on lowering glucose concentrations in mice when compared with acarbose.

Potency ^a^	Compounds	Fold of Lowering Glucose Concentration	
		Dosage (mg/(kg·B.wt))	Fold ^b^
	Acarbose	17.0	1.0 ± 0.6
I	Curcumin	0.3	206.0 ± 12.3 ^c^
II	Antroquinonol	1.0	37.6 ± 2.8 ^c^
	HCD	3.0	17.6 ± 3.4 ^c^
	Docosanol	4.0	15.5 ± 3.8 ^c^
	Tetracosanol	4.0	10.9 ± 0.9 ^c^
III	Rutin	4.0	7.2 ± 2.9 ^c^
	Actinodaphnine	5.0	6.4 ± 2.5 ^c^
	Catechin	6.0	4.4 ± 1.4 ^c^
IV	Quercetin	60.0	1.2 ± 0.2 ^d^
	Berberine	100.0	0.7 ± 0.2 ^d^

The data was represented as mean ± SD (*n* = 5). ^a^ The potency is categorized by comparing with reference drug acarbose. ^b^ The fold change is calculated basis on the efficacy of acarbose set as 1. ^c^ *p <* 0.05 when compared with acarbose. ^d^ *p >* 0.05 when compared with acarbose.
